# Large scale synthesis of copper nickel alloy nanoparticles with reduced compressibility using arc thermal plasma process

**DOI:** 10.1038/s41598-021-86776-0

**Published:** 2021-04-07

**Authors:** Subrat Kumar Das, Arkaprava Das, Mattia Gaboardi, Simone Pollastri, G. D. Dhamale, C. Balasubramanian, Boby Joseph

**Affiliations:** 1 Atmospheric Plasma Division, Institute for Plasma Research, Gandhinagar, 382016 India; 2 Elettra-Sincrotrone Trieste, S.S. 14, Km 163.5 in Area Science Park, 34149 Basovizza, Italy; 3 Homi Bhabha National Institute, Anushakti Nagar, Mumbai, 400 094 India

**Keywords:** Nanoscale materials, Materials science, Nanoscale materials

## Abstract

Among the various methods employed in the synthesis of nanostructures, those involving high operating temperature and sharp thermal gradients often lead to the establishment of new exotic properties. Herein, we report on the formation of Cu-Ni metallic alloy nanoparticles with greatly enhanced stiffness achieved through direct-current transferred arc-thermal plasma assisted vapour-phase condensation. High pressure synchrotron X-ray powder diffraction (XRPD) at ambient temperature as well as XRPD in the temperature range 180 to 920 K, show that the thermal arc-plasma route resulted in alloy nanoparticles with much enhanced bulk modulus compared to their bulk counterparts. Such a behaviour may find an explanation in the sudden quenching assisted by the retention of a large amount of local strain due to alloying, combined with the perfect miscibility of the elemental components during the thermal plasma synthesis process.

## Introduction

For the past nearly three decades, the field of nanomaterials has attracted large interest among both the fundamental science researchers as well as technology developers. This largely owes to exotic properties exhibited by materials when broken down to nano dimensions. To cite some examples, metal nanoparticles like silver and gold have shown significant antibacterial and antimicrobial properties; with silver nanoparticles known to prevent bacterial growth^1^. Gold nanoparticles proved to be effective as targeted drug delivery agents as well as for tumour detection through Surface Enhanced Raman Spectroscopy (SERS)^2^. Alloys and alloyed nanoparticles also hold a large application potential, as they possess synergetic properties derived from each constituent element. For example, studies^3–7^ show that alloying of Pt with other transition metals (like Fe, Co, Ni, Cu, etc.) substantially enhance the electro catalytic performance.

Among various methods for synthesizing alloy nano powders^8–12^, thermal plasma processes represent an ideal route for large scale production^13–16^. Copper-Nickel alloy nano powders were chosen in this study owing to their wide range of applications from magnetic hyperthermia^10,17^, catalytic application in water–gas shift reaction^18^, pyrolysis of ammonium perchlorate^19^, etc. Cu and Ni together form an isomorphous system as they show complete solubility within each other in both solid and liquid phases. In addition, copper and nickel together form a substitutional solid solution with no structural changes occurring due to alloying. In this system, with variation in composition, the free energy change (Δ*G)*, enthalpy change (Δ*H),* and entropy change (Δ*S)* also vary in a quite simple manner.

Stergar et al*.*^8^ report on the preparation of Ni_0.725_Cu_0.275_ with a particle size of 3–10 nm by micro emulsion technique. The particles were found to be super paramagnetic with a controlled Curie point. The authors have also pointed out the requirement for a thermal homogenization of the synthesized nano particles in the NaCl matrix. However, the process also yielded NiCuO paramagnetic impurities. Liu et al*.*^20^ reports on the preparation of Cu-Ni nano spheres by a one-pot chemical synthesis process, showing the easy control of both size as well as elemental ratio of the product against the precursor ratio. Morphological, crystallographic, and stability aspects of the nanoparticles are also reported. However, XRD peaks presented by the authors were asymmetric in shape, most likely as a result of compositional in-homogeneity at the atomic level, given the low temperature synthesis process. Solanki et al*.*^21^ reports on the preparation of both Ni–Co and Ni–Fe by sol–gel method and successfully obtained nanoparticles in the size range of 10–20 nm, exhibiting lower coercivity as compared to their bulk phases. The elemental ratios of both Ni–Co and Ni–Fe were maintained at 1:1. These authors also report on the structure and morphology of the obtained nanoparticles. Santos et al*.*^22^ reports on the use of proteic sol–gel technique for the preparation of Fe–Ni alloy nanoparticles, obtaining a size range of 10–40 nm and concluding that the products have thermal stability against oxidation up to 250 °C.

Hirayama and Takagi^23^ report on the compositional homogeneity of Fe–Co alloy nanoparticles prepared by induction thermal plasma technique. The average particle size is reported to be 90 nm and a saturation magnetization (*M*_*s*_) close to the bulk value. These authors obtained some deviation in the composition ratio between the precursor and the product. Song et al*.*^13^ reports on the use of arc plasma evaporation for preparation of Ni–Cu nanoparticles similar to the present work. They have obtained particle sizes of ~ 50 nm and also found the composition ratio of the product nanopowder being different from that of the precursor in most of their studies. Raut et al*.*^24^ have used thermal plasma and prepared Fe–Ni alloy nanoparticles with varying precursor ratios. This study highlighted changes in the crystal structure as well as thermal stability of the oxidation reaction. In short, non-thermal plasma process is reported to produce controllable and smaller particle sizes as well as compositional ratios. Thermal plasma process, on the other hand, is reported to produce a larger particle size and a compositional ratio different from the feed material ratio^13^.

The present work reports on the use of arc plasma process for the synthesis of Ni–Cu nanoparticles. We also highlight the enhanced mechanical strength/compressibility of these nanoparticles by comparing these with corresponding elemental nanoparticles prepared by other synthesis routes. Alloy nanoparticle preparation by thermal plasma process is challenging and, to the best of our knowledge, only very few reports are available^13,23,24^. As mentioned earlier, thermal plasma synthesis of alloy materials may result in very different alloy composition than the feed material. Different thermo-physical and transport properties of individual precursor material leads to different rate of evaporation, diffusion and condensation. This effectively leads to the formation of a product with different stoichiometry in comparison to the precursor ratio used. In this work an attempt is made to evaluate feasibility of synthesizing homogeneous and single phase Cu-Ni alloy nanoparticles by transferred arc thermal plasma.

Generally, micro structural properties are considered while referring to bulk materials. But with a reduction in size to the nano scale, surface area increases drastically, which, together with interface properties, play a dominant role in the overall enhanced properties of the nanomaterial. The present work brings to the fore two important aspects: (1) the feasibility of forming chemically homogeneous binary alloy nano powders by thermal plasma process and (2) improved mechanical properties under compressive loading. While the chemical homogeneity of the alloy nanoparticles is required to trigger relevant catalytic behaviour; good mechanical properties and sinter-ability are the key requirements of miniature component fabrication by powder metallurgy route.

## Materials and methods

Cu-Ni binary alloy nano powders have been synthesized by vapour phase condensation brought about by thermal plasma process. Initially, an alloy ingot was prepared using high purity Copper (99.7% and 325 mesh, Loba Chemie, product No: 02949) and Nickel powders (~ 99.51% and 200 Mesh, Loba Chemie product No.04860) mixed in the proportion Cu:Ni :: 0.05:0.95. This powder mixture was arc melted in Helium atmosphere and at slightly higher pressure of approximately 2 bar. Nickel concentration in the raw material was maintained at a higher ratio as Ni has low saturation vapour pressure in comparison to copper for all temperatures. Composition of the prepared alloy ingot was confirmed to be 0.05:0.95 by ICP-OES. Prepared alloy ingot was then placed in a graphite anode crucible of 2 inches diameter and 1 inch depth. Another graphite rod of 10 mm diameter was used as cathode. The pictorial reperesentation of the experimental set up is shown in Figure S1 of the electronic supplementary information (ESI) file. The experimental procedure starts with evacuating the synthesis chamber and achieving a base vacuum of 5 × 10^−2^ mbar and then flushing it with Helium gas until the chamber reaches atmospheric pressure. This was done to create an inert gas environment and to avoid oxide formation. An IGBT based regulated DC power supply was used as a current source for generating arc plasma. Arc voltage of 40 V and arc current of 100 A were applied between the electrodes for evaporating the raw materials. Arcing was done for a duration of 7 min. Chamber walls and flanges were cooled by flowing water at 20 °C to maintain a stable thermal gradient. The evaporated material leaves the hot plasma zone encountering a sharp temperature drop. This fall in temperature leads to a burst of nucleation sites and subsequent growth of nano clusters. These nano clusters, or nanopowders, get deposited onto the inner wall and flange surfaces to be later collected, by manually scraping the powders off from the surface and moving them into sample containers. The collected nano powders were used for further studies and analyses.

A fraction of the collected nanopowder sample was dispersed in isopropyl alcohol and drop coated on a Formavar coated copper grid for TEM (FEI Tecnai G2 300 kV) analysis for determining the size and shape of the nanoparticles. Elemental composition analyses were done by EDX to estimate the relative atomic percentage.

Room-to-high temperature synchrotron X-ray powder diffraction (XRPD) data (λ = 0.774354 Å) were collected at the MCX beamline at the Elettra Sincrotrone in Trieste, Italy. A standard quartz capillary of 100 μm diameter was filled with nano powders and evacuated connecting to a vacuum pumping system. High temperature XRPD measurements were carried out in dynamic vacuum thus avoiding oxidation. Diffraction patterns were acquired in Debye geometry on a 4-circle Huber goniometer. A capillary prepared in the same manner but sealed using a butane torch was used for XRPD measurements within the temperature range 100–400 K at the XRD1 beamline (λ = 0.7 Å) of Elettra Sincrotrone, where a Pilatus2M detector was used for the data collection and an Oxford cryocooler (model 700) was used for temperature control. Cu and Ni *K*-edge X-ray absorption spectra were collected at the XAFS beam line of the Elettra Sincrotrone. The X-ray source was routed through a double crystal Si (111) monochromator and the spectra were recorded in transmission mode. The energy calibration for all the samples was done by recording simultaneously a reference spectra of a metal (Ni or Cu) foil placed in a second experimental chamber after the sample and first ionization chamber. Energy was calibrated from the foil spectra using the first inflection point at the absorption edge. All spectra were collected at ambient temperature and with an energy step width of 5 eV for the first 200 eV, 0.2 eV for the near-edge region and a *k*-constant step of 0.03 Å^−1^ in the subsequent higher energy region (EXAFS part). At least two spectra were collected for all samples and were then carefully merged ensuring a perfect overlap, with the intent to increase the signal to noise ratio. The spectra were normalized with the EXAFS part of the spectra.

High pressure (HP) diffraction studies were carried out at the Xpress beamline^25^ of the Elettra Sincrotrone. A monochromatic X-ray beam source of wavelength 0.4957 Å was used for this study. For the HP diffraction measurements a gear driven plate diamond anvil cell from Almax with culets size of 550 μm was used. The high pressure measurements were carried out in a 200 μm stainless steel foil which was indented to 70 μm with a through hole of diameter 180 μm in the middle, prior to loading of the diamonds. Silicone oil was added as pressure transmitting media. The actual pressure experienced by the nanoparticles was determined by standard ruby fluorescence method. XRPD patterns were recorded using a large 2D MAR345 image plate detector.

## Results and discussion

**Figure 1:**
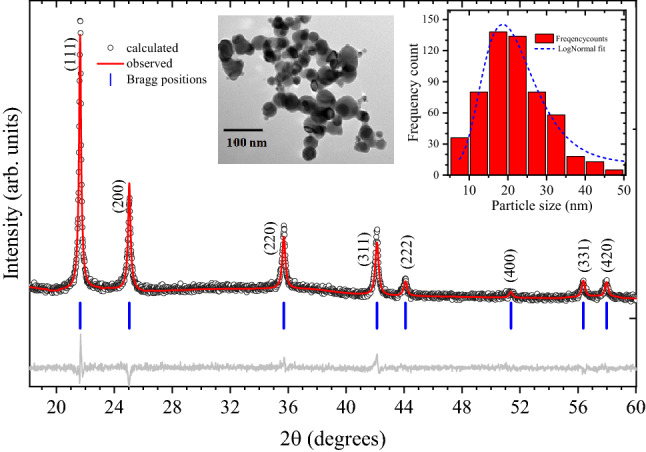
X-ray powder diffraction pattern (XRPD) of the samples at 50 °C, measured with λ = 0.77435 Å; Symbols are measured data, solid redline is the refinement result, grey solid line is the difference pattern and vertical blue bars are the calculated Bragg peak positions. Inset here shows a typical TEM image and nanoparticle size distribution extracted by analyzing several of such images.

Transmission Electron Microscopy images (see inset in Fig. 1) indicate that the obtained nanoparticles are 10–40 nm in size with an average size of 22 nm. Particle size distribution is also shown in the inset of Fig. 1, obtained by analysing 574 clearly distinguishable particles from various micrographs of the same sample. Spot EDX results indicate that the composition of the nano powders is ~ 40:60 (Ni:Cu) with a mean deviation of 2.17%. About 130 randomly selected nanoparticles were taken for EDX analysis giving the compositioinal distribution of individual particles as shown in Figure S2 (ESI). The fractional atomic percentage for each of the elements present in the alloy has been collected from 15 different locations and presented in Table S1 (ESI). The average Ni to Cu ratio in the nanoparticles is significantly different from the starting bulk concentration which is basically due to differences in the thermo-physical and transport properties of the two constituents. Copper and nickel exists in liquid form below 2840 and 3005 K respectively. Process of nucleation for binary alloy metal particles is more complex than that of the single metal precursors as the process is sensitive to the thermo-physical and transport properties of the individual component metal specie. Differences in the evaporation rate, vapour pressure, diffusion rate, condensation rate etc., of individual species either independently or in mixed phase may lead to variations in the final composition. In the studies by Kanhe et al*.*^26^, no significant difference was seen in the initial and final composition of Fe–Ni alloy while it was observed for Al-Ni alloy. The reason was attributed to the different vapor pressure of individual species. For Fe–Ni system, both metals have nearly same vapor pressure and diffusion coefficients thus found an overlapping super-saturation regions. In the present scenario, vapor pressure of copper is only slightly higher than that of nickel, 2.06 bar for copper and 1.01 bar for nickel at 3000 K^27,28^. However, their thermal conductivities are much different. At room temperature, for copper, it is 397 W/(m–K) and 88 W/(m–K) for nickel. This would result in copper reaching its boiling point before nickel. This results in earlier evaporation of copper in Cu-Ni mixture leading to its supersaturation and start of nucleation. Therefore, Cu nucleates before Ni by homogenous gas phase condensation process. However, since Nickel concentration in feed material is high (95%), a considerable amount Ni atoms also vaporise when the temperature reaches its boiling point. During collisions Cu atoms serve as nucleating site for Ni and vice-versa. This may likely lead to the formation of core shell nanoparticles; however, we do not observe any such features in the TEM images: Only non-faceted spherical particles are seen. This confirms that at high temperature, the two elements diffuse into each other due to diffusion and leading to the formation of alloys^26^. Apart from the thermo-physical properties of evaporating materials, the process of gas phase nucleation and growth of particles in thermal plasma environment is also dependent on the gas cooling rate at the periphery of plasma where the temperature is suitable for nucleation to occur (< 3500 K approximately)^29^. In the present scenario, cooling rate near the anode surface is ~ 10^6^ K/s and the energy density of plasma is ~ 10^8^ J/m^3^ as obtained from simulation results. The simulation results regarding the plasma temperature distribution and cooling rate profile have been shown in Figure S3(a,b) of ESI.

Synchrotron XRPD data also confirm the alloy composition to be Cu_60_:Ni_40_ with a cubic lattice parameter (3.57678(24) Å at 30 °C) falling systematically between those of pure copper (3.597 Å) and nickel (3.499 Å)^30^. Figure 1 shows the XRPD data together with the results of the Rietveld refinement. A careful look at the data reveals that the diffraction peaks are broader compared to the silicon standard used for calibration, which is in accordance with the nanometric size of the studied powder samples. Profile shape analysis carried out considering a Scherrer-like peak broadening (proportional to $\cos^{ - 1} \left( \theta \right)$) for spherical particles results in an average domain size of about 32(2) nm. A slight improvement is achieved after the introduction of an extra term of broadening to simulate the microstrain, resulting in a δd/d ≈ 0.5% at 30 °C. The latter, proportional to $\tan \left( \theta \right)$, reflects the non-ideal randomization of the two metals occupying the same lattice site^31^. This is commonly observed in partially disordered systems^32^. There is a fair agreement between the model and the data (R_wp_ ~ 7.9%; goodness of fit = 1.3), thus confirming the phase purity of the synthesized nanoparticles.

**Figure 2:**
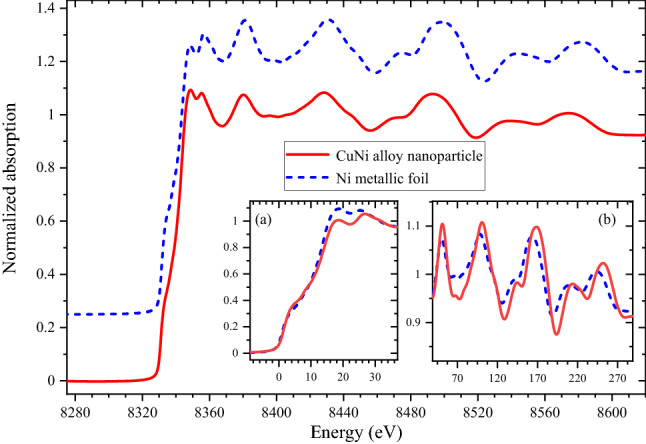
Normalized X-ray absorption fine structure (XANES) spectra at Ni K-edge of the sample (red curve) and metallic Ni foil (blue curve). Insets (**a**) and (**b**) respectively show zoomed areas over the edge and the near-edge features without any vertical shift unlike in the main panel where the two spectra are shifted vertically for clarity in presentation. For the insets, the absorption edge energy is set to zero.

Nickel *K*-edge X-ray absorption near edge spectrum (XANES) shows the metallic nature of the sample indicating the absence of oxidation during the synthesis process. XANES features at Ni *K*-edge coincide well with that of the Ni foil, with minor differences which can be understood as due to the alloying effect coupled to the nano-size of the grains. In particular, there is an increased intensity for the pre-edge feature [see inset (a) in Fig. 2] combined with a decreased intensity in the whiteline (8348.7 eV). The near-edge features [see inset (b)] show similar features as the metallic Ni foil, but for a small shift towards higher energy which is consistent with identical local environment with a small change in the lattice parameters and hence the bond distances in the two systems.

**Figure 3:**
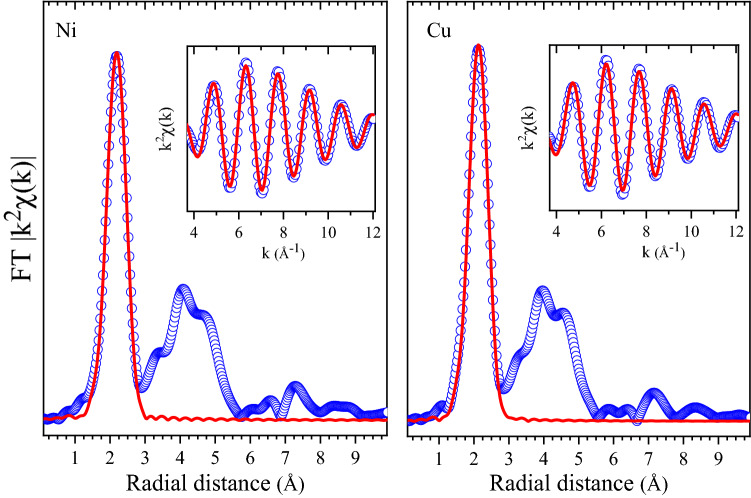
Fourier transform (FT) magnitudes of the Ni and Cu K-edge EXAFS (symbols) together with the results of the first shell modelling (solid lines). Insets shows the filtered EXAFS oscillations (weighted by k^2^) corresponding to the first shell. In the insets, symbols are experimental data and solid lines are the model.

Extended X-ray absorption fine structure (EXAFS) data at both Ni and Cu *K*-edges were utilized to investigate the local structure around two metal components in the sample. The EXAFS data from both edges reveal very similar features indicating comparable local structure. The Fourier transforms (FT) magnitudes from the Ni and Cu *K*-edges are shown in Fig. 3. The structure of the FT magnitudes is fairly matching up to 5.5 Å, which underlines the occurrence of a perfect alloying, thus avoiding any local clustering of the metallic species in the nanoparticles. Above 5.5 Å, the EXAFS data is heavily influenced by the noise level. As can be noted from the EXAFS equation^33^, the FT magnitude is inversely proprotional to the square of the radial distance. In this case with increasing radial distance, above 5.5 Å, the noise level becomes significant to the signal level to discuss meaningfully the differences seen the Ni and Cu *K*-edge FT magnitudes as seen in Fig. 3.

**Figure 4:**
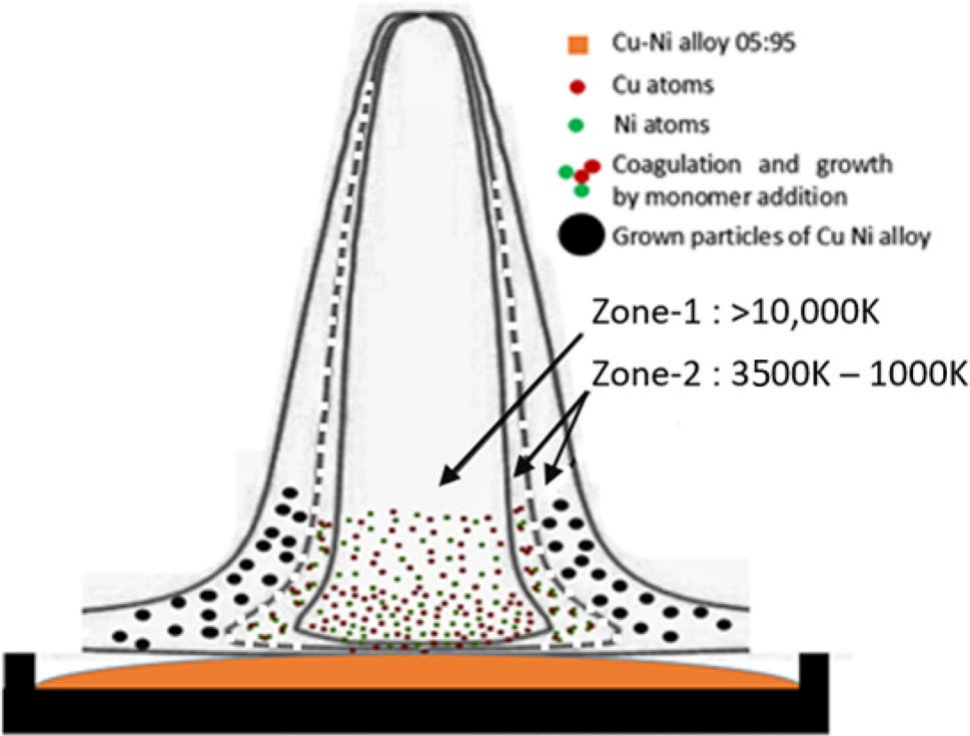
Schematic representation of thermal plasma process leading to the nucleation and growth of nanoparticles by vapour phase condensation. (See also the schematic diagram of the arc-plasma set up shown in Fig . S1).

We now briefly describe the alloy nanoparticle formation process. A cartoon drawing showing schematics of the nanoparticle formation (nucleation and growth) by vapour phase condensation method is presented in Fig. 4. In arc thermal plasma route, zone 1 is the plasma column, wherein the temperature is very high (~ 10,000 K) and the nucleation or nanoparticle formation is prohibited. Cluster formation commences from constituent vapors near the plasma-metal interface where the temperature is ≤ 3500 K which is the inner part of zone 2 (Fig. 4). When the vapor is being transported to zone 2, cluster formation via nucleation and monomer addition takes place with increasing population density. With further drop in temperature in the outer part of zone 2, clusters start to act as nucleation sites and begin to grow. Subsequent cooling while moving further away from the plasma column results in formation of nanoparticle at the outside of zone 2^29^. Further growth is restricted due to reduced temperatures (< 1000 K) existing beyond zone 2. The above schematics and the stated temperatue values take inputs from our own plasma simulations (see details in ESI). Therefore, outer zone 2 is responsible for formation of the alloy nanoparticles.

Nearly spherical-shaped particles or non-faceted particles are observed in both SEM and TEM. This could be due to homogeneous nucleation by vapour to liquid transformation occurring through monomer addition^26^. To enable a better appreciation of the nanoparticle morphology a few more TEM micrographs at different scale are shown in Figure S4 (ESI).

Thermodynamically, Cu-Ni forms a weakly miscible solid solution and has a bulk miscibility gap extended up to 630 K^34^. The synthesis of nanoparticles above miscibility temperature (MT) leads to chemical homogeneity whilst the synthesis below this temperature leads to formation of binary alloy particles. Thermal plasma route of synthesis involves an operating temperature which is higher than the miscibility gap temperature. Copper and Nickel have a very small lattice mismatch and a small positive enthalpy of mixing. Bochicchio et al*.*^35^ reports that the segregation effect can be minimized in case of smaller sizes. Guisbiers et al*.*^36^ suggest that the miscibility gap temperature decreases with the reduction in size and the number of facets. The simulated miscibility temperature for Cu-Ni alloy with 5 nm and 10 nm side edge length and 20 facets of polyhedral is around 450 K and 580 K respectively which will keep on increasing with increasing particle diameter^36^. In the present scenario, the particle diameter value is around 22 nm considering non faceted spherical shape. So, it can be anticipated that MT would be more than 580 K but less than the MT for bulk—which is 630 K. The temperature in zone 2 is 1000 K which is much higher than MT and hence it is expected that the solution would be completely miscible and no segregation would happen. The operating temperature during nucleation and growth in plasma route synthesis process falls within 3500 K to 1000 K which is much higher than MT which validates the condition for alloy formation. In zone 2, highly homogeneous clusters get rapidly quenched at a rate, ~ 10^6^ K/sec well as from reported literature^37^. Hence, the distribution remains intact as the process is too fast for re-ordering or precipitation. All these features facilitate the formation of highly homogeneous alloy nanoparticles.

Having discussed the nanoparticle formation mechanism, let us come back to the discussion of their microscopic characterization. The EXAFS data have been analysed considering the first-shell, which include the near-neighbour Ni–Cu and Ni–Ni in case of Ni *K*-edge and Cu–Cu and Cu–Ni bond distances in case of Cu *K*-edge. The filtered EXAFS data together with the results of the first shell modelling, considering the two types of bonds, are shown in the insets of Fig. 3. These results are in agreement with the 60:40 compositions extracted from EDX and XRPD. However, EXAFS model results indicate that local Cu–Ni bonds are only slightly different than the Cu–Cu or Ni–Ni bonds. Such a deviation of the local bond distances from what is expected from Vegard’s law has been observed in several systems^38–40^. This is also reflected in the extra broadening of the XRPD pattern, modelled by the microstrain term added to the profile shape.

**Figure 5:**
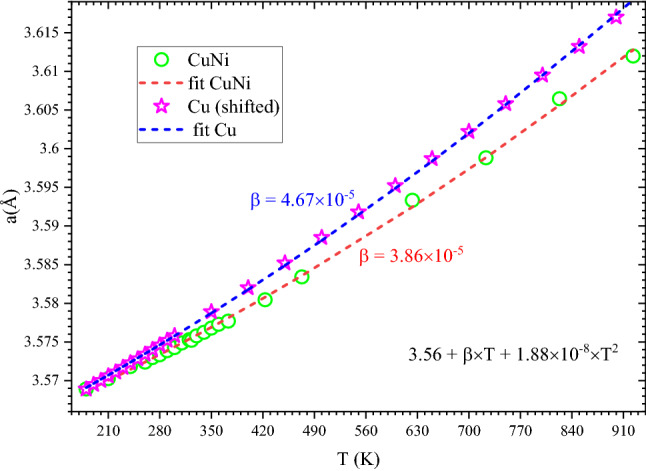
Comparison between temperature dependences of CuNi nanoparticles (circles) and bulk Cu, [(^41^)stars] lattice parameters. For ease in comparison, copper lattice parameters are shifted down by a constant (0.0396 Å). Dashed lines are 2nd order polynomial fit to the data where the β values are indicated in the graph.

Temperature dependent diffraction data from 180 to 920 K were used to investigate the lattice parameter evolution with temperature. These results are shown Fig. 5 and reveal a regular thermal expansion of the system, which can be well described by a 2nd order polynomial 3.56 + β × *T* + 1.88 × 10^−8^ × *T*^2^, where β = 3.86 × 10^−5^ Å/K. For a ready comparison, we have also plotted the temperature dependence of the bulk Cu lattice parameter reported in the literature with a constant *y* shift of 0.0396 Å. For the bulk copper, the above polynomial describes the temperature dependence with β = 4.67 × 10^−5^ Å/K, larger than that of Cu-Ni nanoparticles. Both systems show a regular lattice expansion with temperature; however, the linear term in the alloy nanoparticle is smaller revealing a stiffer lattice.

**Figure 6:**
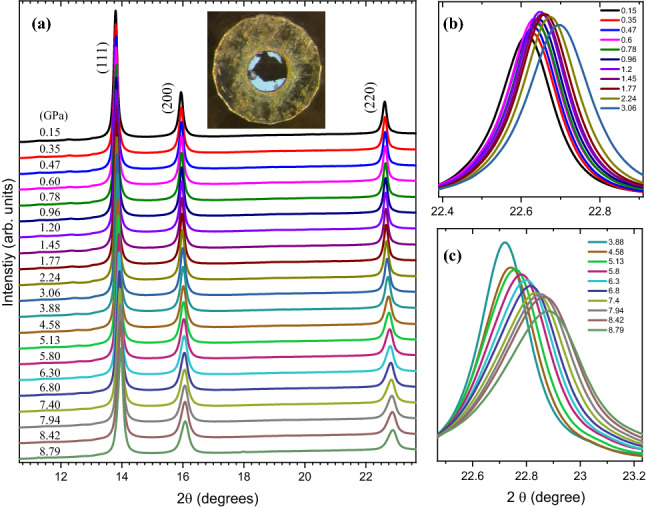
Pressure dependent XRPD data. Panel (**a**) shows the full pattern; panels (**b**) and (**c**) show a zoom into the 220 peak without any vertical shifts. Pressure values are shown in the legends of panels (**b**) and (**c**) with identical colour codes as in (**a**). Inset in (**a**) shows the Cu-Ni sample (black) together with a two ruby balls (white spheres) in the sample chamber (inner circular region). Diameter of the outer circular metallic container is 500 μm, identical as the culet size of the diamonds.

To further understand the structural properties of the obtained Cu–Ni alloy nanoparticles, high pressure X-ray powder diffraction (HP-XRPD) measurements were undertaken. HP-XRPD data as a function of pressure up to ~ 9 GPa is shown in Fig. 6. Data indicate non-occurrence of any phase transition except a regular reduction in unit-cell parameters (Fig. 6b,c) as a response to the applied pressure.

**Figure 7:**
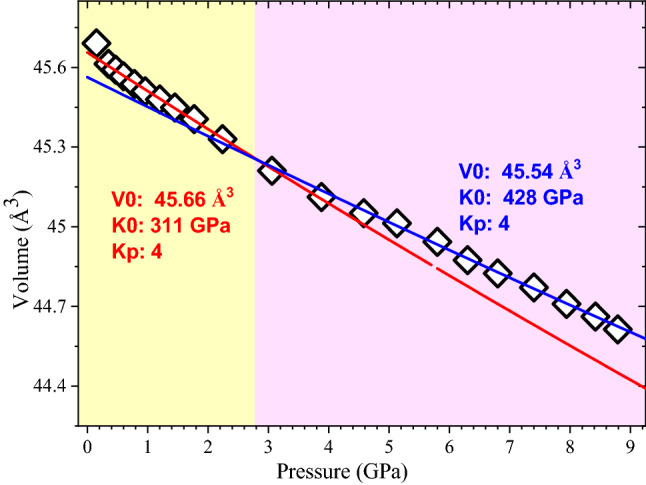
Pressure dependence of the unit-cell volume. Symbols are experimental data. Solid lines are fit to a second order Birch-Murnaghan equation. Obtained parameters of the equation of state (E_0_S–V_0_ is extrapolated zero pressure unit-cell volume. K is the bulk modulus; Kp is the pressure derivative of K) are also indicated. In the fit shown (solid lines) Kp is kept constant to 4.

In Fig. 7, we present the pressure dependence of the unit-cell volume to applied pressure. A second order Birch-Mrunaghan (BM) equation with *Kp*, the pressure derivative of the bulk modulus *K*, fixed to four, is found to describe the data below 3 GPa for *V*_0_ = 45.66 Å and *K* = 311 GPa. The pressure dependence of the unit-cell volume above 3 GPa is found to fall on similar second order equation with *V*_0_ = 45.54 Å and *K* = 428 GPa. The *K* values obtained are rather high compared to the bulk modulus observed for Ni nanoparticles^42–44^. The highest value reported is 228 GPa for 20 nm Ni nanoparticles^42^. Compared to this, we observe *K* = 311 GPa, in the low-pressure regime. This enhancement is found to be 136%. If we consider the bulk modulus we observe in the high-pressure regime above 3 GPa, which is 428 GPa, this enhancement is 188%. In some cases, a larger *K* value is observed for nano systems compared to corresponding bulk^45^. For example, in case of Au nanoparticles Hong et al., found *K* value to be 196 GPa, about 17% higher than the corresponding bulk where it is 167 GPa^46^. There were also reports on even higher enhancement of bulk modulus; for example in Au systems, Gu et al., observed *K* value to be 286 GPa, much higher than that of the corresponding bulk^47^. In a similar fashion, the *K* value we obtained are rather high, which demonstrate a large stiffness of the DC thermal plasma synthesized alloy nanoparticles. This unusual low compressibility could be a result of substitutional solid solution that brings further strengthening of the materials. Alloys like Copper-Nickel, Copper-Zinc etc., wherein the atoms of constituents are similar and are uniformly distributed are known to form such substitutional solid solution with the resulting strengthening of the system^48,49^. The high temperature plasma process results in same level of the fluid phase transport of copper and nickel atoms and the sharp temperature gradients (fast quenching) ensure that this distribution remains and segregation of atoms does not occur during the nanoparticle formation.

## Conclusion

Chemically homogeneous Cu-Ni binary alloy nano powders of size range 10–30 nm were successfully synthesized by thermal plasma based gas phase condensation method. Both X-ray diffraction and EXAFS confirmed a nearly stoichiometric alloy formation, thus underlining the capability of the thermal plasma based gas phase condensation method to extend the nanoparticle synthesis process to mixed metallic alloys. High Temperature XRPD has highlighted a lower linear thermal expansion compared to non-alloyed metals. High pressure diffraction showed that the nanoparticles have higher strength (or lower compressibility) than their constituent elements Cu and Ni, which has been attributed to the substitutional solid solution brought about by high temperature miscibility and retention of this phase due to fast quenching in the arc-plasma process. Such properties can be useful for applications which require lower compressibility compared to the pure metallic nanoparticles. The yield of alloy nanopowders from our experiments is few tens of milligrams in span of 7 min). However the process is easily scalable in addition to it being an instantaneous and single step. These characteristics of thermal plasma process are highly conducive for industrial scale production of alloy nanopowders.

## Supplementary Information

Supplementary Information.
