# Supplementary material for: Large scale synthesis of copper nickel alloy nanoparticles with reduced compressibility using arc thermal plasma process
Source: Sci Rep. 2021 Apr 7;11:7629. doi: 10.1038/s41598-021-86776-0 (PMC8026964; doi:10.1038/s41598-021-86776-0)
Supplement: Supplementary file 1 — Supplementary Information. [file 41598_2021_86776_MOESM1_ESM.docx]

Electronic supplementary Information

**Large scale synthesis of copper-nickel alloy nanoparticles with reduced compressibility using arc thermal plasma process**

Subrat Kumar Das^1^, Arkaprava Das^1^, Mattia Gaboardi^2^, Simone Pollastri^2^, G.D. Dhamale^1^,C. Balasubramanian^1,3*^ and Boby Joseph^2*^

*^1^Atmospheric Plasma Division, Institute for Plasma Research, Gandhinagar, 382016, India*

*^2^Elettra-Sincrotrone Trieste, S.S. 14, Km 163.5 in Area Science Park, Basovizza 34149, Italy*

*^3^Homi Bhabha National Institute, Anushakti Nagar, Mumbai – 400 094. India*

**Experimental setup of arc plasma**


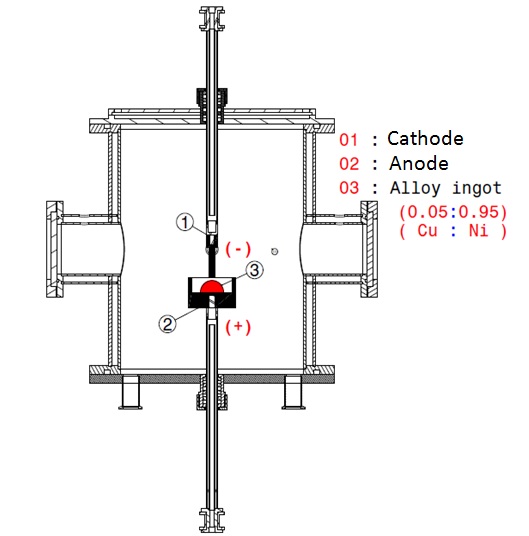


**Figure S1**: Schematic of the experimental setup for synthesis of the alloy nanoparticles

The experimental set up used for preparation of Cu-Ni alloy nanoparticles consists of a water cooled double walled SS chamber with ports for cathode and anode (*Details of the experiments are provided in Page 4 of the manuscript*)


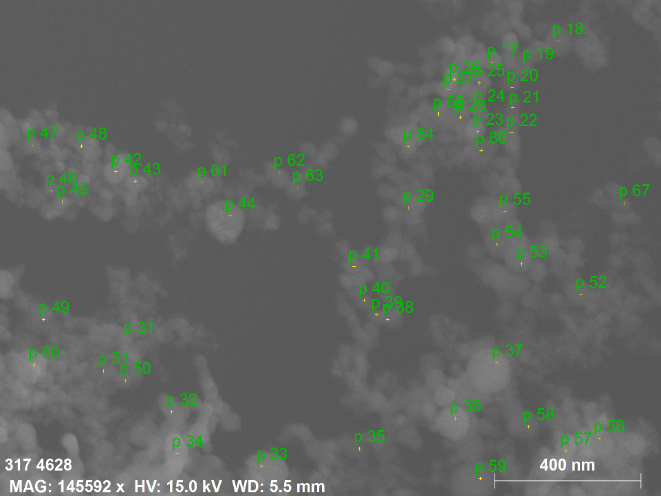


**Figure S2**: The above SEM image indicates the locations of 66 points (marked in green) wherein compositional measurements by EDAX was collected. This analysis was done on other SEM images as well and a total of 130 particles were analysed for the composition. The panel on right shows the statistical distribution for all the 130 points (*Reference to this analysis is given in Page 5 of the manuscript*)

**Table S1:**Atomic percent (%)estimated from EDX spectra of 15 pointsshown in the SEM image above (ESIF-2). (*Reference to this table details are in Page 5 of the manuscript*)

| **Spectrum** | **Ni%** | **Cu%** |
| --- | --- | --- |
| 1 | 40.37 | 59.63 |
| 2 | 40.11 | 59.89 |
| 3 | 40.83 | 59.17 |
| 4 | 40.30 | 59.70 |
| 5 | 39.64 | 60.36 |
| 6 | 40.72 | 59.28 |
| 7 | 41.70 | 58.30 |
| 8 | 39.24 | 60.76 |
| 9 | 39.02 | 60.98 |
| 10 | 39.91 | 60.09 |
| 11 | 39.62 | 60.38 |
| 12 | 38.83 | 61.17 |
| 13 | 40.59 | 59.41 |
| 14 | 39.27 | 60.73 |
| 15 | 40.78 | 59.22 |
| Mean value | 40.06 | 59.94 |
| Sigma | 0.79 | 0.79 |
| Sigma mean | 0.72 | 0.72 |

**Figure S3**: The plasma temperature distribution (a) and cooling rate profile (b) near (2mm above) the anode surface. The above results were obtained from CFD simulation using FLUENT© commercial software. The simulation was carried out for DC-transferred arc plasma operated at 4kW (100A × 40V) of input DC power in helium environment at 1atm pressure. The output voltage obtained from simulation is 37V which is closely matching the experimental value i.e. 40V. (*This data is used in the manuscript for estimation of temperature gradient*)

**Figure S4:** Additional TEM micrographs for Cu-Ni alloy nanoparticle showing non-faceted nearly spherical shaped particles at different scales. (*Refer to the text in Page 10 of the manuscript*)
